# Exome sequencing identifies *SLC26A4*, *GJB2*, *SCARB2* and *DUOX2* mutations in 2 siblings with Pendred syndrome in a Malaysian family

**DOI:** 10.1186/s13023-017-0575-7

**Published:** 2017-02-21

**Authors:** Yock-Ping Chow, Nor Azian Abdul Murad, Zamzureena Mohd Rani, Jia-Shiun Khoo, Pei-Sin Chong, Loo-Ling Wu, Rahman Jamal

**Affiliations:** 1 UKM Medical Molecular Biology Institute (UMBI), Chancellor Tuanku Muhriz Hospital, UKM Medical Centre, Jalan Yaacob Latif, 56000 Cheras, Kuala Lumpur, Malaysia; 2 Codon Genomics S/B, No 26, Jalan Dutamas 7, Taman Dutamas, Balakong, 43200 Seri Kembangan, Selangor Malaysia; 3 Department of Pediatrics, Chancellor Tuanku Muhriz Hospital, UKM Medical Centre, Jalan Yaacob Latif, 56000 Cheras, Kuala Lumpur, Malaysia

**Keywords:** Pendred syndrome, Exome sequencing, Syndromic hearing loss, Childhood deafness, Autosomal recessive inheritance

## Abstract

**Background:**

Pendred syndrome (PDS, MIM #274600) is an autosomal recessive disorder characterized by congenital sensorineural hearing loss and goiter. In this study, we describing the possible PDS causal mutations in a Malaysian family with 2 daughters diagnosed with bilateral hearing loss and hypothyroidism.

**Methods and Results:**

Whole exome sequencing was performed on 2 sisters with PDS and their unaffected parents. Our results showed that both sisters inherited monoallelic mutations in the 2 known PDS genes, *SLC26A4* (ENST00000265715:c.1343C > T, p.Ser448Leu) and *GJB2* (ENST00000382844:c.368C > A, p.Thr123Asn) from their father, as well as another deafness-related gene, *SCARB2* (ENST00000264896:c.914C > T, p.Thr305Met) from their mother. We postulated that these three heterozygous mutations in combination may be causative to deafness, and warrants further investigation. Furthermore, we also identified a compound heterozygosity involving the *DUOX2* gene (ENST00000603300:c.1588A > T:p.Lys530* and c.3329G > A:p.Arg1110Gln) in both sisters which are inherited from both parents and may be correlated with early onset of goiter. All the candidate mutations were predicted deleterious by in silico tools.

**Conclusions:**

In summary, we proposed that PDS in this family could be a polygenic disorder which possibly arises from a combination of heterozygous mutations in *SLC26A4, GJB2* and *SCARB2* which associated with deafness, as well as compound heterozygous *DUOX2* mutations which associated with thyroid dysfunction.

**Electronic supplementary material:**

The online version of this article (doi:10.1186/s13023-017-0575-7) contains supplementary material, which is available to authorized users.

## Background

Hearing loss is a multifactorial disease with nearly 50% of cases being heritable and attributable to genetic defects [1]. The annual incidence of congenital deafness is estimated to be 1:1000 newborns [2], hence, identification of the heritable causal genes is pivotal to reduce the incidence of childhood deafness. Pendred syndrome (PDS, MIM #274600) is among the most common types of syndromic hearing impairment, and accounts for approximately 10% of hereditary deafness [3]. PDS is clinically characterized by sensorineural deafness, enlargement of the vestibular aqueduct (EVA), goiter, and incomplete iodide organification [3, 4]. Even though PDS could be clinically confirmed with perchlorate discharge test in combination with temporal bone scan of the ear architecture, misdiagnosis with other deafness associated disease remains as the key challenge [5]. Late onset of goiter manifestation which usually develops after the age of 10 years and the presentation of only mild hypothyroidism have made definitive diagnosis of PDS difficult [6, 7]. Furthermore, the degree of hearing loss could vary from mild to profound, either contributed by physical malformation or genetic defects. The limitations to getting an accurate diagnosis will prevent early treatment and may lead to mental retardation which is preventable by thyroxine replacement therapy [8]. As such, identification of genes contributed to PDS is desirable to pave the way towards early detection of PDS as well as for carrier testing.

PDS is a complex genetic disease which may be inherited monogenically or digenically [4, 9–11]. It has been well documented that biallelic mutations in *SLC26A4* (MIM #605646) is the hallmark of PDS, with a frequency of 25% [4, 9]. Clinically, *SLC26A4* mutation has been used as genetic test to differentiate between PDS and non-syndromic familial EVA, which otherwise would not be possible to clinically distinguish, even with perchlorate discharge test [6, 12]. However, nearly 50% probands harboured only monoallelic mutation in *SLC26A4*, and for some patients, PDS is not due to S*LC26A4* gene mutations [4]. The discovery of the involvement of other deafness genes, including *FOXI1* (MIM #601093), *KCNJ10* (MIM # 602208) and *GJB2* (MIM #121011) [9–11] in combination with *SLC26A4* monoallelic mutation has proposed the existence of digenic inheritance pattern in PDS and EVA. The complexity of the genetic defects attributed to PDS suggests that a comprehensive mutational screening is warranted to identify the disease causal genes.

In the past, limitations in genomic sequencing technologies have only permitted the identification of disease-causing mutations through the candidate gene screening approach. Now, with the advent of next generation sequencing technologies, genome wide screening can now be performed in a cost-effective manner. Among these, whole exome sequencing (WES) is preferable as it focuses only on coding regions in which ~85% disease-causing mutations are located [13]. WES has also successfully discovered genes for many rare diseases [14]. Given that the genetic makeup of PDS remains largely unknown and complex, we performed WES to identify the genes responsible for PDS in a family with 2 affected siblings and their unaffected parents. This study will enhance our understanding about the genetic aetiology underpinning PDS, and to identify candidate genes which may be useful for precise molecular diagnosis and to guide family planning for better management of heritable deafness.

## Methods

### Subjects

Two siblings diagnosed to have PDS were referred for molecular evaluation and confirmation of diagnosis. These sisters were the only children of a pair non-consanguineous parents. They were 15 and 9 years old respectively at the time of referral.

### Elder sister

The elder sister first presented to her local doctor at the age of 10 months with progressively enlarging goiter. Investigation then showed hypothyroidism and L-thyroxin was started. At 3 years of age, her parents noted profound hearing impairment for which she required hearing aid. There was no other significant past medical or surgical history and she was not on any long term medications. Her parents reported that she had normal speech development and was able to attend normal school. On examination, she had a large, diffused multinodular goiter. Ultrasound of the thyroid gland revealed multiple complex cysts. Tc99m thyroid scan showed a hyperfunctioning multinodular goiter with increased total iodine uptake of 34.6%. These findings were consistent with dyshormonogenesis (goitrous hypothyroidism with increased radioactive iodine uptake by the thyroid). Unfortunately, perchlorate discharge test could not be done. Hearing test confirmed bilateral profound sensorineural hearing loss > 60 dB, worse for high frequency sounds. Magnetic resonance image (MRI) of the inner ear and temporal bones showed bilateral dilated vestibular aqueduct and presence of only 1 ½ turns of the cochlear (normal: 2 ¾ turns) consistent with cochlear hypoplasia. In view of the large goiter size, patient underwent total thyroidectomy at 13 years of age. Currently she is receiving full dose of thyroxin i.e. 100 mcg daily.

### Younger sister

The younger sister presented with profound hearing loss at 10 months of age associated with delayed speech development. At 16 months, she was wearing hearing aid and receiving speech therapy. She underwent cochlear implantation at 5 years of age. Her parents also noticed progressively enlarging goiter then. She had delayed speech development. She used sign language for communication and attended special school. On examination, there was a large, diffused multinodular goiter. Ultrasound revealed multiple complex cysts within the thyroid gland. Tc99m thyroid scan showed a hyperfunctioning multinodular goiter with increased total iodine uptake of 30.4%. Hearing test showed bilateral profound sensorineural hearing loss > 60 dB, worse for high frequency sounds. MRI of the inner ear and temporal bones showed bilateral dilated vestibular aqueduct and presence of only 1 ½ turns of the cochlear (normal 2 ¾ turns) consistent with cochlear hypoplasia. Total thyroidectomy was anticipated.

### Exome library construction and sequencing

Peripheral blood samples were collected from all the individuals included in this study with written informed consent. Genomic DNA was extracted from peripheral blood using salt extraction method and the DNA quality was assessed using agarose gel electrophoresis. The DNA samples were of good quality (A260/A280 > 2.0; A260/A230 > 2.0) as assessed by Nanodrop (Thermo Fisher Scientific, USA). The DNA concentration was measured using Qubit dsDNA BR Assay Kit (Thermo Fisher Scientific, USA). The DNA libraries were prepared employing the Ion AmpliSeq™ Exome RDY Kit (Thermo Fisher Scientific, USA) and were then sequenced by the Ion Proton™ System (Thermo Fisher Scientific, USA), according to the manufacturer’s protocol.

### Bioinformatic data analysis

Read mapping and variant calling were performed by the Ion TorrentSuite™ v4.4.2 software (Thermo Fisher Scientific, USA) using default parameters setting. The reads were aligned to human reference genome hg19, followed by variant calling using TorrentSuite™ Variant Caller v4.4.2.1. Next, the variants with SNP quality scores ≤ 30 were filtered out using SnpSift [15], followed by annotation with ANNOVAR [16]. Only non-synonymous variants in the coding regions (exonic, splicing) with a read depth greater than 5X were retained for further analysis. Polymorphisms with allele frequencies > 0.01 reported in 1000 Genomes Project, NHLBI Exome Sequencing Project, and Maximum Population Frequency were filtered out. Subsequently, we identify the candidate disease causing mutation by comparing the variants detected in affected sisters with their parents based on monogenic (autosomal recessive), followed by digenic and polygenic inheritance traits. Variants which fulfilled the above criteria were manually inspected using Integrative Genomics Viewer to filter out false positive variants [17, 18]. The effect of the variants was assessed using several in silico prediction tools, including SIFT [19], Polyphen2 [20], MutationTaster [21], FATHMM [22], CADD [23], PROVEAN [24], and DANN [25]. Candidate mutations which predicted deleterious by one of the above tools were further studied by searching literature database.

### Sanger validation

A total of 5 predicted pathogenic candidate mutations, i.e. *SLC26A4* (ENST00000265715:c.1343C > T, p.Ser448Leu), *GJB2* (ENST00000382844:c.368C > A, p.Thr123Asn), *SCARB2* (ENST00000264896:c.914C > T, p.Thr305Met), *DUOX2* (ENST00000603300:c.1588A > T, p.Lys530*), and *DUOX2* (ENST00000603300:c.3329G > A, p.Arg1110Gln) were selected for validation by Sanger sequencing. The primers were designed using Primer3 (Additional file 1: Table S4). The regions were amplified by PCR using AmpliTaq Gold Polymerase (Thermo Fisher Scientific, USA), and the amplified products were purified using PCR Purification Kit (Qiagen, Germany), and sequenced using ABI BigDye Terminator v3.1 Cycle Sequencing Kit (﻿Thermo Fisher Scientific, USA﻿). The chromatograms were visualized using BioEdit software.

## Results

### Whole-exome sequencing

We sequenced the exomes of 4 individuals from a family with 2 daughters diagnosed with PDS and their unaffected parents. An average of 36 million reads were generated per sample, and the reads were mapped to the human reference genome hg19, with 91% of the bases covered at > 20X coverage. The mean depth of coverage was 104X with a uniformity of 91%. The variants were filtered as described in the Materials and Methods, and the summary of the sequencing results are as shown in Table 1.

**Table 1:** Results of exome sequencing of 2 affected sisters and their unaffected parents

Parameter	Elder sister	Younger sister	Father	Mother
Mapped reads	42766143	24025346	37859563	40082904
On-target	95.42%	95.48%	96.65%	96.62%
Mean coverage	120.5X	68.36X	111.5X	117X
20X coverage	92.97%	85.20%	91.95%	93.72%
Uniformity	90.95%	91.24%	90.68%	92.24%
No. of total variants	53753	52397	52433	53316
No. of total variants with quality score ≥ 30	51907	49251	51016	51941
No. of coding variants	21276	20200	20839	21059
No. of nonsynonymous variants	10703	10156	10454	10563
After removal of polymorphisms	796	797	731	688
Shared Candidate PDS mutations				
Homozygous	1	1	0	0
Compound heterozygous	2	2	0	0
Heterozygous inherited from father	132	132	132	NA
Heterozygous inherited from mother	121	121	NA	132
Candidate PDS mutations in elder sister only				
Homozygous	0	NA	NA	NA
Compound heterozygous	5	NA	NA	NA
Heterozygous inherited from father	73	NA	73	NA
Heterozygous inherited from mother	83	NA	NA	83
Candidate PDS mutations in younger sister only				
Homozygous	NA	0	0	0
Compound heterozygous	NA	3	0	0
Heterozygous inherited from father	NA	80	80	NA
Heterozygous inherited from mother	NA	73	NA	73

### Identification of disease causing genes in Pendred syndrome

After filtering out polymorphisms with allele frequency > 0.01 as reported in the 1000 Genomes Project, 6500 NHLBI exome and Maximum Population Frequency databases, we retained only non-synonymous variants for identifying PDS causative mutations. Subsequently, we filtered the variants based on monogenic autosomal recessive trait. Variants which present in both sisters were prioritized for further investigations. Our analysis identified *DUOX2* (MIM #606759) compound heterozygous mutations (ENST00000603300: p.Lys530X, p.Arg1110Gln) were inherited by both sisters from their father and mother respectively, and may be causative to goiter manifestation. However, we did not find additional homozygous or compound heterozygous mutations which may be contributed to the deafness phenotype.

By taking into consideration that PDS could be inherited via digenic trait, we then look into heterozygous mutations which were inherited by both sisters from their unaffected parents. Interestingly, both sisters inherited missense mutations in 2 genes which known to be associated with PDS (i.e. *SLC26A4:* ENST00000265715:c.1343C > T,p.Ser448Leu; *GJB2:* ENST00000382844:c.368C > A, p.Thr123Asn) from their father, as well as another deafness gene, *SCARB2* (ENST00000264896:c.914C > T,p.Thr305Met) from their mother. These 3 candidate mutations were predicted pathogenic by one of the variant effect prediction tools (i.e. SIFT, Polyphen-2, MutationTaster, FATHMM, CAAD, PROVEAN and DANN; Additional file 2: Table S1 and Additional file 3: Table S2), hence we postulated that the combination of these 3 candidate missense mutations may be contributed to PDS and associated with hearing loss phenotype. It is noteworthy to mention that *SCARB2* could be a novel PDS candidate gene and needs further investigation.

Furthermore, we also looked into the autosomal recessive mutations which are present only in either probands. As depicted in Table 2, each sisters harboured additional non-shared compound heterozygous mutations, however none of these candidate genes are known to be implicated in PDS. Further analysis on heterozygous mutations (Additional file 2: Table S1 and Additional file 3: Table S2) found that the elder sister harboured additional mutations which may be contributed to the PDS phenotype (Additional file 4: Table S3), including hearing (inherited from father: *DIAPH3, GPR171, LOXHD1*; inherited from mother: *MCOLN3, SYNE4*), and thyroid function (inherited from father: *C16orf89*; inherited from mother: *TXNDC11*). However, these additional mutations have not exaggerated the manifestation of the hearing loss or goiter, in which both sisters were diagnosed with bilateral hearing loss and presented with goiter during 1 year old. Hence, we postulated that these additional mutations which present only in elder sister may not be relevant to PDS.

**Table 2:** List of homozygous and compound heterozygous variants detected in this study

Mutation	Mutation Type	Elder sister	Younger sister	Father	Mother
Mutations shared by two affected siblings					
*LDB3*: ENST00000429277:c.1384C > A:p.Pro462Thr	Hom	AA	AA	CA	CA
* DUOX2*: ENST00000603300:c.1588A > T:p.Lys530*	CompHet	TA	TA	TA	TT
*DUOX2*: ENST00000603300:c.3329G > A:p.Arg1110Gln	CompHet	CT	CT	CC	CT
*COL22A1*: ENST00000303045:c.923A > G:p.Glu308Gly	CompHet	TC	TC	TC	TT
*COL22A1*: ENST00000303045:c.2101A > G:p.Met701Val	CompHet	TC	TC	TT	TC
Mutations detected in elder sister only					
*TECPR2*: c.1477C > T:p.Pro493Ser	CompHet	CT	CC	CT	CC
*TECPR2*: c.1457C > T:p.S486Leu	CompHet	CT	CT	CC	CT
* CHRD*: c.2261C > G:p.Ala754Gly	CompHet	CG	CC	CG	CC
*CHRD*: c.993G > C:p.Gln331His	CompHet	GC	GG	GG	GC
*CYP4V2*: ENST00000378802:c.237G > T:p.Gln79Asp	CompHet	GT	GG	GT	GG
* CYP4V2*: ENST00000378802:c.367A > G:p.Met123Val	CompHet	AG	AA	AG	AA
* CYP4V2*: ENST00000378802:c.780C > G:p.Ile260Met	CompHet	CG	CG	CC	CG
*RAPGEF6*: ENST00000509018:c.664C > T:p.Arg222Cys	CompHet	GA	GG	GA	GG
*RAPGEF6*: ENST00000509018:c.1963A > G:p.Thr655Ala	CompHet	TC	TT	TT	TC
* MUC22*: ENST00000561890:c.2998_299AC	CompHet	GA/AC	GA_HOM	GA-HOM	GA/AC
*MUC22*: ENST00000561890:c.3441_3442AG	CompHet	CA/AG	CA/AG	CA/AG	CA_HOM
*MUC22*: ENST00000561890:c.3493A > C:p.I1165Leu	CompHet	AC	AC	AC	AA
Mutations detected in younger sister only					
*CR1*: ENST00000367049:c.7310 T > C:p.Leu2437Pro	CompHet	TC	TC	TC	TT
*CR1*: ENST00000367049:c.6919G > A:p.Gly2307Arg	CompHet	GG	GA	GG	GA
*MUC16*: ENST00000397910:c.38825A > G:p.Gln12942Arg	CompHet	TT	TC	TC	TT
*MUC16*: ENST00000397910:c.11458A > G:p.T3820Ala	CompHet	TT	TC	TT	TC
*MUC16*: ENST00000397910:c.7201G > T:p.Ala2401Ser	CompHet	CC	CA	CA	CC
* FRAS1*: ENST00000264895:c.1471C > T:p.R491Trp	CompHet	CT	CT	CC	CT
* FRAS1*: ENST00000264895:c.11893A > G:p.Asn3965Asp	CompHet	AA	AG	AG	AA

*Abbreviations*: *Hom* homozygous, *CompHet* compound heterozygous

### Sanger validation

**Fig. 1:**
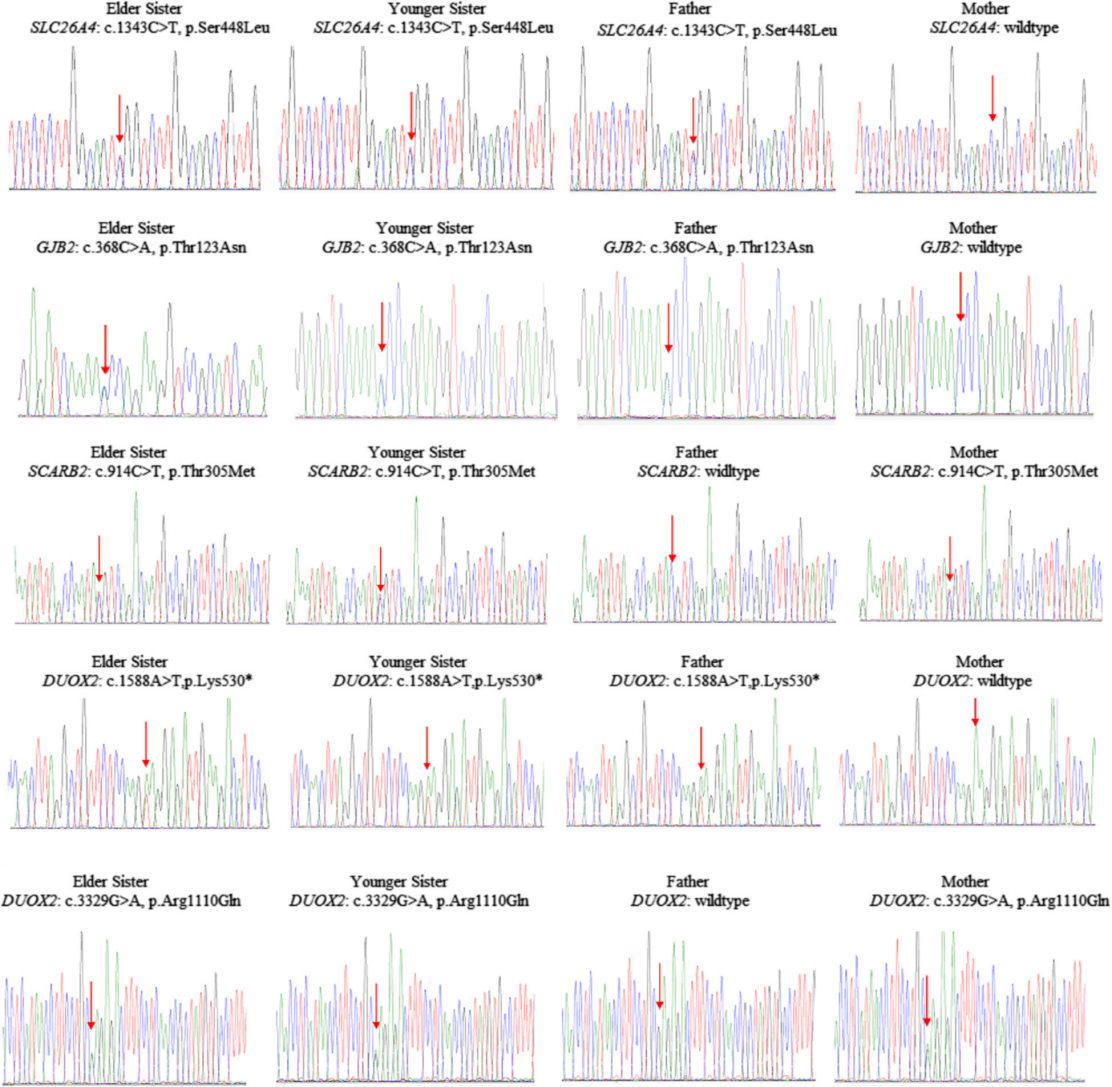
Sanger validation of *DUOX2* compound heterozygous mutations (ENST00000603300:c.1588A > T:p.Lys530* and c.3329G > A:p.Arg1110Gln), *SLC26A4* (ENST00000265715:c.1343C > T, p.Ser448Leu), *GJB2* (ENST00000382844:c.368C > A, p.Thr123Asn), and *SCARB2* (ENST00000264896:c.914C > T, p.Thr305Met) heterozygous mutations. The arrow shows the site of the changes

Candidate genes predicted to be associated with PDS were selected for further validation by Sanger sequencing. Mutations which were present in both sisters and father (*SLC26A4:* c.1343C > T, p.Ser448Leu; *GJB2:*c.368C > A, p.Thr123Asn; *DUOX2:* c.1588A > T, p.Lys530*), and present in both sisters and mother (*SCARB2:* c.914C > T, p.Thr305Met; *DUOX2*: c.3329C > A, p.Arg1110Gln) were confirmed. The chromatograms are depicted in Fig. 1.

**Fig. 2:**
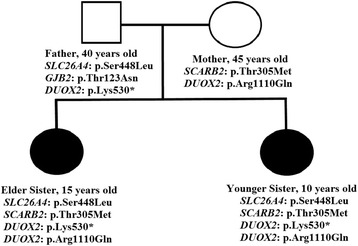
Pedigree of the family with autosomal recessive Pendred syndrome. Open symbols denote unaffected parents; filled *black* symbols denote affected siblings

As summarized in Fig. 2, the father was a heterozygous carrier for *SLC26A4, GJB2* and *DUOX2,* whereas mother was a heterozygous carrier for *SCARB2* and *DUOX2*. Both of the sisters harboured the same heterozygous mutations in *SLC26A4, GJB2 and SCARB2*, hence suggesting that the combination of these 3 heterozygous mutations may led to hearing loss in these patients. Furthermore, *DUOX2* compound heterozygous mutation may be associated with early onset of hypothyroidisms and goiter.

## Discussion

Clinically, PDS is characterized by the manifestation of a combination of severe to profound sensorineural hearing loss, inner ear anomalies such as Mondini’s dysplasia, EVA or vestibular anomalies, and goiter [26–28]. Also, deafness in PDS generally profound (>60 dB) with prelingual onset [29], and sometimes a fluctuating but worsening course [30–32], consistent with a progressive lesion of the sensory organ. In this case study, clinical diagnosis confirmed both sisters were PDS: (1) MRI examination of the inner ear confirmed both sisters had EVA, an essential prerequisite for the diagnosis of PDS [33, 34]; (2) both sisters had bilateral sensorineural hearing loss, with frequency > 60 dB; (3) both sisters are euthyroid and diagnosed with hypothyroidism at age of 1 year old; (4) the disease is potentially heritable via autosomal recessive or digenic/polygenic traits as both sisters are affected whilst their parents were unaffected.

It has been long considered that PDS is a monogenic disease attributed to *SLC46A4* biallelic mutations [35, 36] or a digenic disease attributed to a combination of *SLC46A4* and *KCNJ10*, *FOXI1* or *GJB2* [9–11]. Notably, our analysis did not detect homozygous or compound heterozygous in the known PDS genes (i.e. *SLC26A4*, *KCNJ10, FOX1*, *GJB2*) based on monogenic autosomal recessive trait, hence suggesting PDS in this family could be a more complex digenic or polygenic disorder. Interestingly, both sisters were found inherited *SLC26A4* and *GJB2* monoallellic mutation from their unaffected father. Loss of function in both *SLC26A4* and *GJB2* have been implicated in syndromic and non-syndromic hearing loss [10, 37, 38]. Whilst *SLC26A4* defects mainly attributed to syndromic hearing loss, *GJB2* mutations accounts for up to 50% of all recessive non-syndromic hearing loss based on ethnic background [39]. Essentially, S*LC26A4* involves in maintaining the endocochlear potential [35, 36], whereas *GJB2* play role in auditory transduction by recycling potassium ions back to the endolymph of the cochlear duct [40]. Given that both genes play pivotal roles in maintaining normal hearing function, we postulated that the *SLC26A4* and *GJB2* missense mutations are among the PDS driver mutations in this family. In addition, in contrast with earlier studies which have shown biallelic mutation of *SLC26A4* to be correlated with bilateral EVA, while monoallelic mutation or zero mutation of *SLC26A4* correlated with unilateral EVA [35, 39, 41], we did not observe the association of this monoallelic *SLC26A4* mutation and the severity of cochlea anomalies. Both sisters with *SLC26A4* monoallelic mutation had incomplete partition type II abnormalities and presented with bilateral hearing loss at the age of 13 and 8 years old respectively.

As the evidence showing *SLC26A4* and/or *GJB2* monoallelic mutation was not sufficient to cause PDS in this family, we explored the implication of other possible causal mutations. Our analysis discovered pathogenic heterozygous mutation in another deafness associated gene, *SCARB2* (MIM #602257), in both siblings and mother. *SCARB2* encodes for lysosomal integral membrane protein type 2, which is involved in membrane transportation and the reorganization of endosomal and lysosomal compartment. An earlier study has shown loss of function in *SCARB2* being implicated in hearing loss, whereby the *SCARB2* knockout mice manifested cochlear deafness, which is associated with massive spiral ganglion neuron losses, concomitant with loss of the inner and outer hair cells and a strongly impaired capacity to generate an endocochlear potential [42]. Beyond that, mutational analysis also identified that *SCARB2* mutation was associated with hearing impairment [43, 44]. Given that both sisters inherited similar *SCARB2, SLC26A4* and *GJB2* mutations from their unaffected parents, our data supports the notion that a combination of these 3 heterozygous mutations may led to bilateral hearing loss in these 2 sisters.

In addition, we detected a compound heterozygous mutation in *DUOX2* (p.Lys530* & p.Arg1110Gln) in both siblings. *DUOX2* encodes for a key enzyme required to generate hydrogen peroxide (H_2_O_2_) which is essential for thyroid hormone synthesis and normal thyroid function [45, 46]. It has been well documented that mutations in *DUOX2* are associated with congenital hypothyroidism [47–51]. For instance, biallelic and triallelic mutations in *DUOX2* are associated with permanent congenital hypothyroidism, whilst mononoallelic mutation caused transient congenital hypothyroidism [49, 51]. Mutation p.Lys530* and p.Arg1110Gln in *DUOX2* were found in patients with transient congenital hypothyroidism [49]. Earlier studies also have shown that p.Arg1110Glu in *DUOX2* reduced H_2_O_2_ production (5–9%, *P* < 0.01), hence contributed towards transient congenital hypothyroidism [48, 52]. Our analysis suggested that the *DUOX2* compound heterozygous mutations in both sisters may be involved in permanent congenital hypothyroidism, and correlated with significant goiter manifestation at a young age. *DUOX2* mutational screening may be useful to detect thyroid dysfunction as compared to perchlorate discharge test, and to differentiate between PDS and other hearing loss diseases.

Taken together, our analysis suggested that PDS in this family could be a complex polygenic disorder which attributed to a combination of 3 heterozygous mutations implicated in deafness-related genes (*SLC26A4*:p.Ser448Leu; *GJB2*:p.Thr123Asn; *SCARB2*:p.Thr305Met), as well as a compound heterozygous mutation implicated in gene associated with thyroid function (*DUOX2*:p.Lys530* & p.Arg1110Gln).

## Conclusions

In summary, our findings showed that exome sequencing has enabled the identification of new candidate causal genes underlying PDS, and suggested that PDS could be a complex heritable polygenic disorder. In this case study, we postulated that a combination of *SLC26A4*, *GJB2* and *SCARB2* heterozygous mutations may be implicated in deafness, whilst *DUOX2* compound heterozygous mutations may be contributed towards thyroid dysfunction. Screening of additional family members and additional PDS cases may be required to strengthen the usefulness of *SLC26A4*, *GJB2*, *SCARB2* and *DUOX2* as candidate diagnostic biomarkers for PDS.

## Additional files

Additional file 1: Table S4. List of PCR primers used. (XLSX 9 kb)
Additional file 2: Table S1. List of non-synonymous heterozygous mutations inherited from father. (XLSX 94 kb)
Additional file 3: Table S2. List of non-synonymous heterozygous mutations inherited from mother. (XLSX 91 kb)
Additional file 4: Table S3. List of other possible PDS causal mutations which uniquely inherited by the eldest sister. (XLSX 11 kb)

## Abbreviations

EVA: Enlarged vestibular aqueduct
H_2_O_2_: Hydrogen peroxide
MRI: Magnetic resonance image
PDS: Pendred syndrome
WES: Whole exome sequencing
